# Evoked responses to note onsets and phrase boundaries in Mozart's K448

**DOI:** 10.1038/s41598-022-13710-3

**Published:** 2022-06-10

**Authors:** Yijing Feng, Robert J. Quon, Barbara C. Jobst, Michael A. Casey

**Affiliations:** 1 Department of Computer Science, Dartmouth College, Hanover, NH 03755 USA; 2 Geisel School of Medicine, Dartmouth College, Hanover, NH 03755 USA; 3 Dartmouth-Hitchcock Medical Center, Lebanon, NH 03756 USA; 4 Department of Music, Dartmouth College, Hanover, NH 03755 USA

**Keywords:** Computational neuroscience, Neurology

## Abstract

Understanding the neural correlates of perception of hierarchical structure in music presents a direct window into auditory organization. To examine the hypothesis that high-level and low-level structures—i.e. phrases and notes—elicit different neural responses, we collected intracranial electroencephalography (iEEG) data from eight subjects during exposure to Mozart’s K448 and directly compared Event-related potentials (ERPs) due to note onsets and those elicited by phrase boundaries. Cluster-level permutation tests revealed that note-onset-related ERPs and phrase-boundary-related ERPs were significantly different at $-150$, 200, and 450 ms relative to note onset and phrase markers. We also observed increased activity in frontal brain regions when processing phrase boundaries. We relate these observations to (1) a process which syntactically binds notes together hierarchically to form larger phrases; (2) positive emotions induced by successful prediction of forthcoming phrase boundaries and violations of melodic expectations at phrase boundaries.

## Introduction

Musical information is organized hierarchically. The processing of individual musical elements such as phrase boundaries and note onsets, is associated with distinct brain regions and neural responses. Understanding the neural correlates of perception of hierarchical structure in music presents a direct window into auditory organization.

The music-theoretic concept of musical structure describes listeners’ segmentation of auditory information into nested hierarchical units of various sizes^1^. Previous work such as Lerdahl and Jackendoff’s *A Generative Theory of Tonal Music*^2^, which was influenced by Bersteins’s *The Unanswered Question*^3^, attempted to model music understanding with the aid of generative linguistics. In principle, the organization in music is similar to human language, where speech is nested recursively into units such as phonemes and words, and extended to phrases and sentences. Ding et al.^4^ have shown a hierarchy of neural processing timescales underlies grammar-based internal construction of hierarchical linguistic structure. Prystauka et al.^5^ reviewed recent studies and summarized the theories linking the oscillatory markers to the processing of hierarchical structure in languages, such as linking beta oscillation to syntactic structure building and linking gamma oscillation to semantic structure building^6,7^. Correspondingly, music consists of notes, chords, themes, and higher-level functional units such as phrases and sections^8^, which occur at quasi-periodic intervals and are marked by changes in melodic theme, harmony, rhythm, and key^9,10^. These higher-level compositional elements underlie audience engagement with the music and are experienced as anticipation of upcoming events. Thus, phrase-level components are regarded as primary functional units in the cognitive processing of music.

To better understand the cognitive processing of complex auditory information, previous studies have investigated neural responses to important structural elements in music by examining event-related potentials (ERPs). Several ERP components that are linked to syntactic violations in language processing have also been observed in music perception. For example, the N400 component is associated with words that are semantically anomalous given the preceding context^11^, and it was also discovered to be elicited in the processing of out-of-key or unexpected notes in familiar melodies^12,13^. The P600 component, which is sensitive to the non-preferred continuation of a sentence^14^, can also be elicited by incongruous elements in musical sequences^15,16^. In addition, the closure positive shift (CPS), an electrical phenomenon that can be detected at the close of a phrase, has been reported to mark prosodic phrase boundaries in both speech^17^ and music^18^. These findings contribute to the understanding of the perception of individual higher-order structural elements in music.

However, it remains unclear how the human brain processes and integrates auditory information at different hierarchical levels with naturalistic music stimuli. Most previous studies extracted musical phrases from simple melodies or manipulated phrase boundaries by note filling—a commonly used technique to generate unphrased control stimuli by filling pauses with musically plausible notes, which do not allow for the investigation of the neural processing of phrase boundaries in naturalistic music perception. Other studies attempted to explore hierarchy in music perception but failed to reveal the neural correlates of higher-order structural elements due to the lack of score-based segmentation of musical stimuli. These studies relied on neural responses to the noncognitive units marked by pauses or bars^19^, which limited their findings to the lower-level perception of music.

To address the gap in understanding the neural correlates of different hierarchical levels of music perception, we analyzed brain responses to naturalistic music with note-onset and phrase-boundary-related ERPs using a cluster-based permutation test, and localized brain structures activated by these different stimulus elements. The current study extends previous work in two ways: (1) it directly compared the neural responses to musical components at different levels, which helps reveal the hierarchical structure in auditory cognition, and (2) it generalized Knösche‘s result^18^ to naturalistic music perception by using naturalistic, i.e. unmodified, musical-phrase stimuli. We hypothesized that low-level and high-level musical structures would elicit distinct neural responses, and that the processing of low-level structures would be associated with lateral temporal brain regions and high-level structures would involve increased activity in frontal brain regions. As such, our study serves as a foundation for understanding brain responses to the hierarchical structure in music perception.

## Results

### Note-onset-related ERPs and phrase-boundary-related ERPs

A total of twelve sessions of Intracranial Stereo-EEG data were collected from eight subjects with refractory epilepsy undergoing intracranial EEG monitoring for the clinical treatment during exposure to the first 90 s of Mozart’s K448.

**Figure 1:**
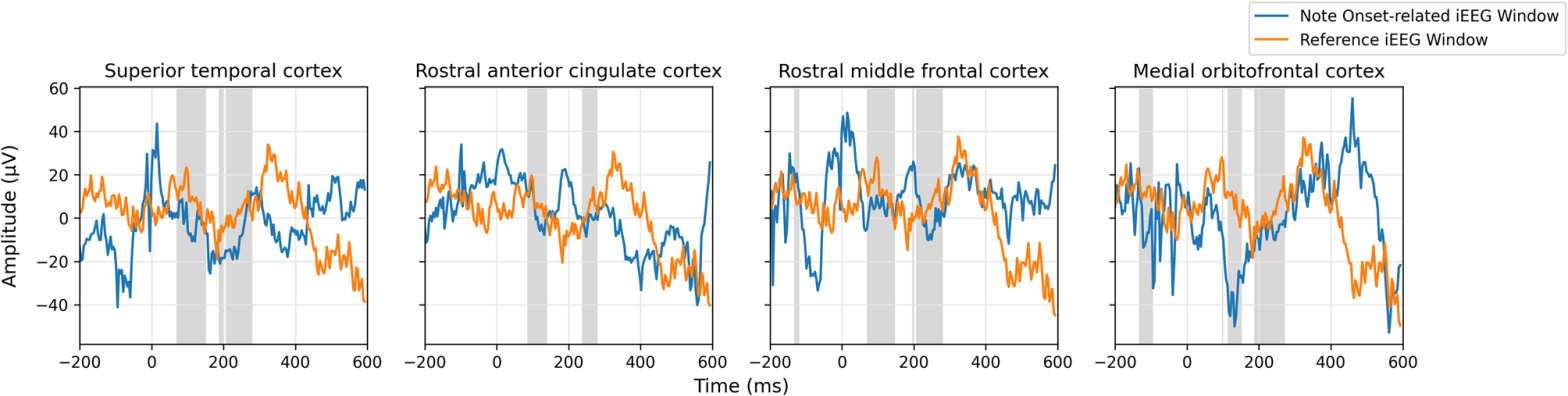
Note-Onset-related ERPs observed at these subregions with significance in at least six sessions are shaded in light gray.

**Figure 2:**
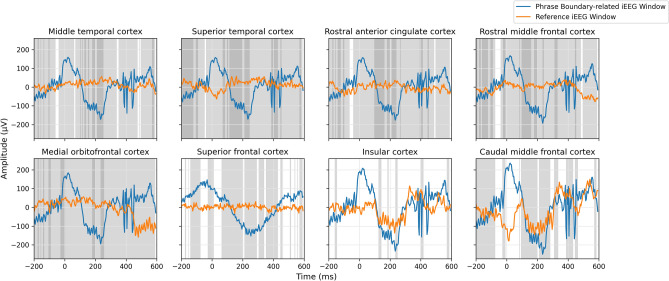
Phrase-boundary-related ERPs observed at these subregions with significance in at least six sessions are shaded in light gray and ten sessions in dark gray respectively.

**Figure 3:**
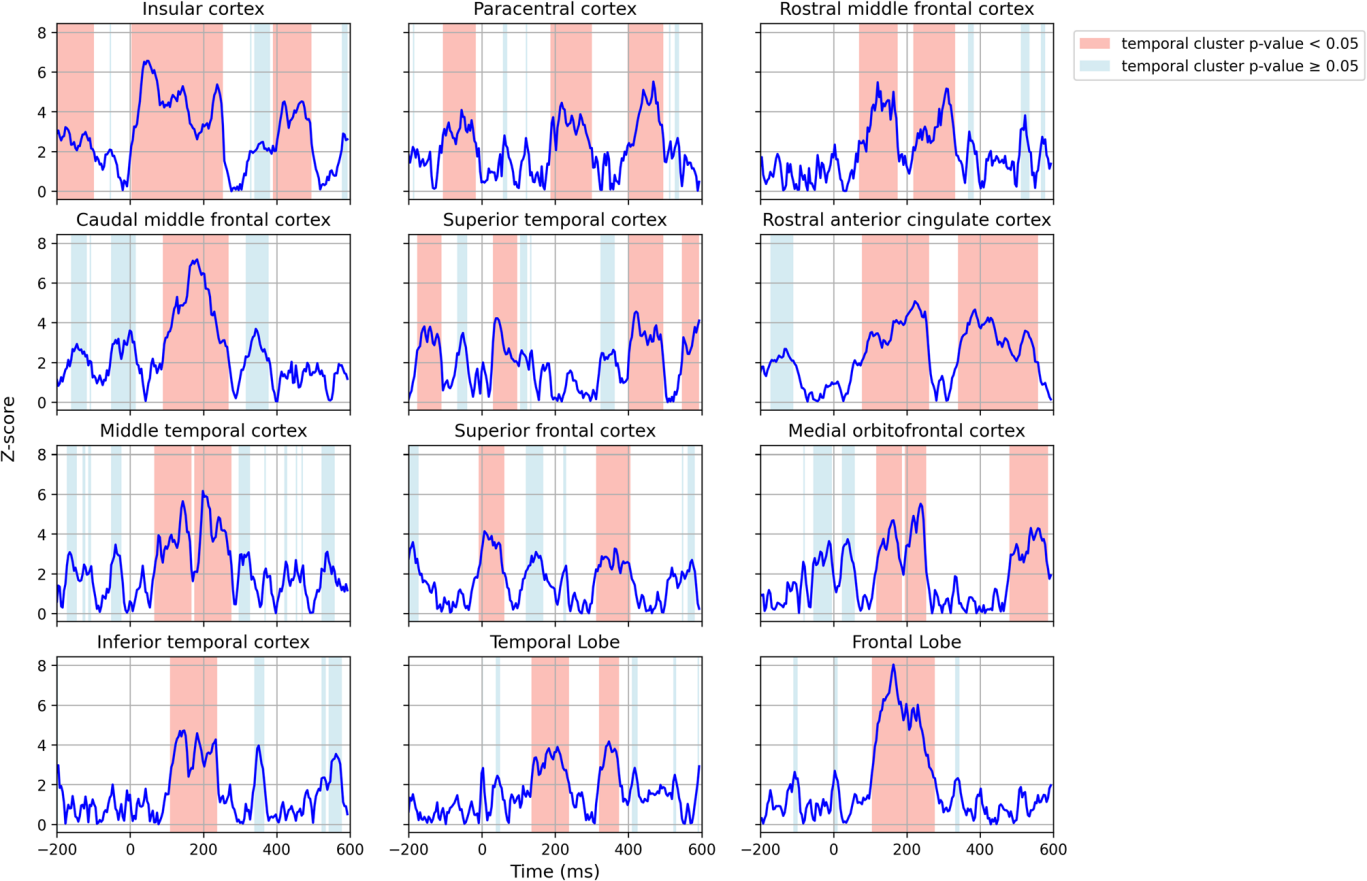
A cluster-based permutation test for each sub-region within a single session (Subject 3, Session 2). The blue line represents the z-score at each time point. The consecutive temporal clusters with z-score $>1.96$ are highlighted. The temporal clusters showing significant ($p<0.05$) difference between the iEEG windows sampled around note onsets and the reference windows are highlighted in red; the non-significant clusters are highlighted in blue.

To verify the hypothesis that both note onsets and phrase boundaries elicit evoked responses, we computed the ERP waveforms (Figs. 1, 2) by averaging intracranial electroencephalography (iEEG) windows sampled near stimulus markers (phrase boundaries and note onsets) across all twelve sessions, and performed a cluster-based permutation test to determine whether the iEEG windows sampled near the stimulus markers were significantly different from reference windows randomly sampled between stimulus markers. Temporal clusters in which a significant difference ($p<0.05$) was observed between the two conditions were reported. The *p*-value statistics of significant temporal clusters are provided in Tables 1 and 2. Figure 3 shows the result of the statistical analysis in each subregion within a single session. We shaded the regions in Figs. 1 and 2 to represent the intersection of significant temporal clusters across all twelve sessions in each subregion.

**Figure 4:**
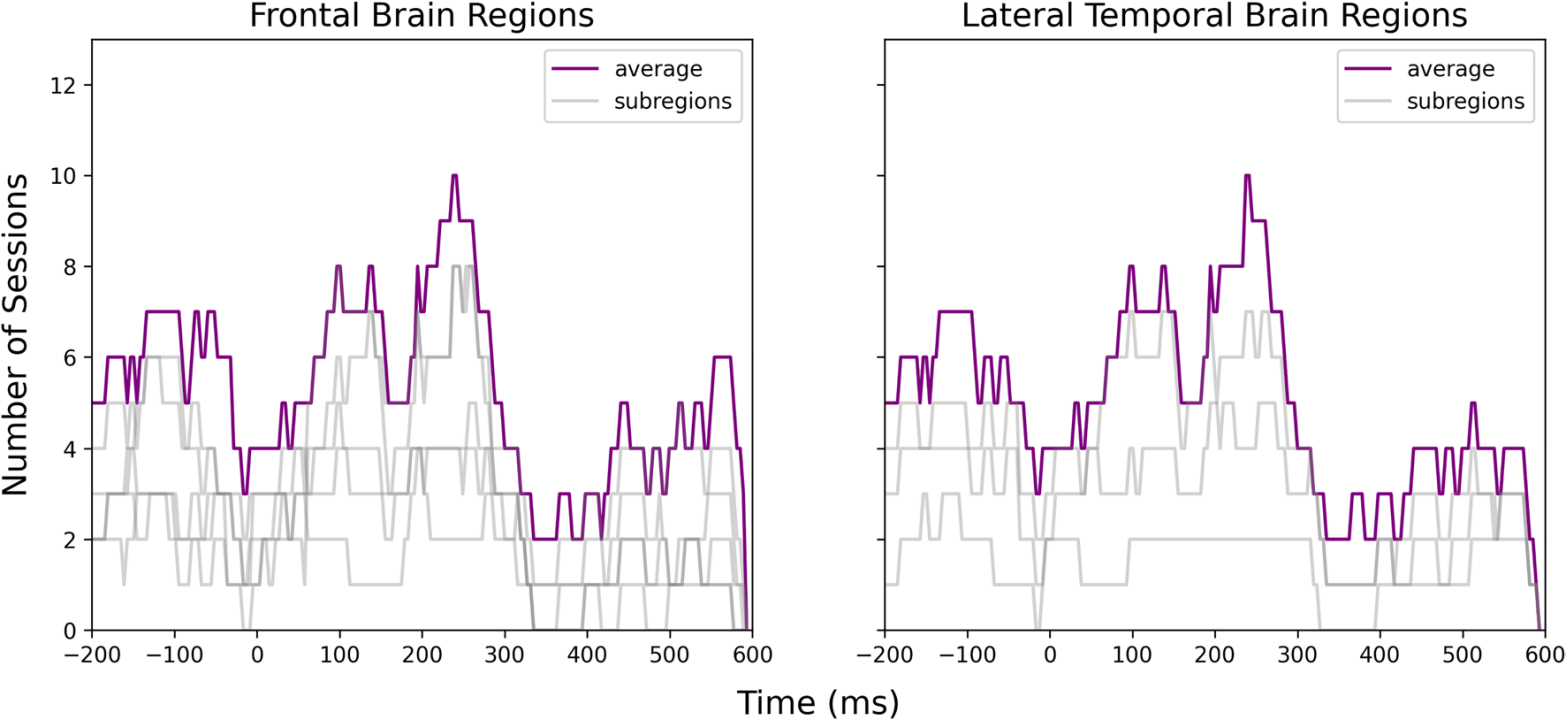
Number of sessions showing significant differences in the iEEG windows sampled around note onsets and the reference windows at each time point. The purple lines were obtained by averaging all channels within the frontal and lateral temporal brain regions. The light gray lines were obtained by averaging the channels within sub-regions that contributed to the frontal and lateral temporal regions.

**Figure 5:**
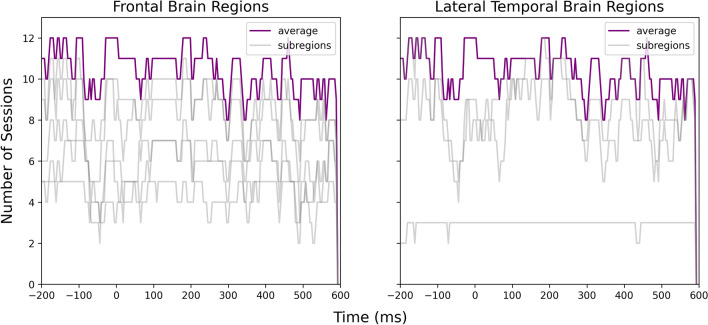
Number of sessions showing significant differences in the iEEG windows sampled around phrase boundaries and the reference windows at each time point. The purple lines were obtained by averaging all channels within the frontal and lateral temporal brain regions. The light gray lines were obtained by averaging the channels within sub-regions that contributed to the frontal and lateral temporal regions.

**Figure 6:**
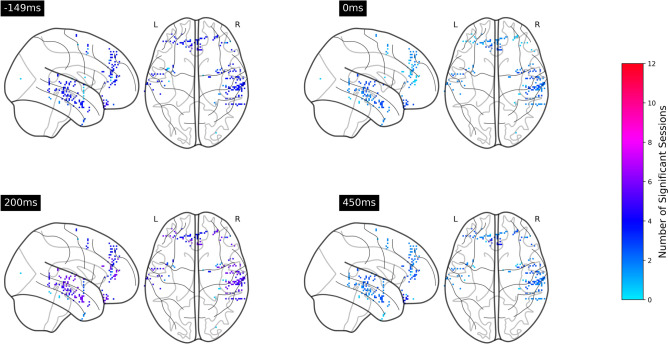
Number of significant sessions within each cortex at $-149$, 0, 200, 450 ms relative to the note onsets. The electrodes were mapped onto a common MNI space. In the integration analysis, although the location of electrodes differed across sessions, all the electrodes within a cortex have identical color for the purpose of visualization .

**Figure 7:**
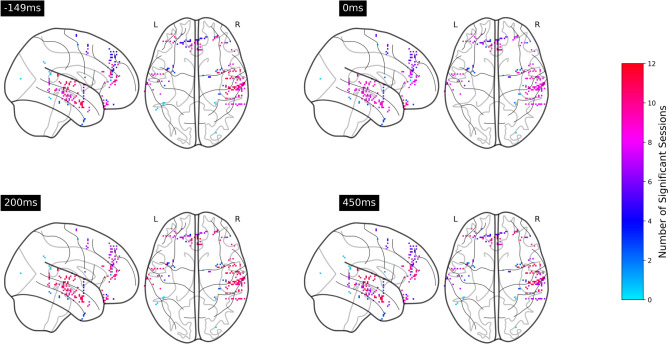
Number of significant sessions within each cortex at $-149$, 0, 200, 450 ms relative to phrase boundaries .

We then integrated the results for frontal brain regions and lateral temporal brain regions over all twelve sessions as shown in Figs. 4 and 5. For note-onset-related ERPs, a majority of sessions had significant temporal clusters at $-150$ and 200 ms around the note onset stimulus markers in both frontal and temporal brain regions. Notably, ten sessions contained significant clusters at around 200 ms. In the analysis of phrase-boundary-related ERPs, all twelve sessions having overlapping clusters at $-150$, 0, 200, and 400 ms around the phrase boundaries in both frontal and temporal brain regions. Figures 6 and 7 illustrate the cortical distribution of the statistical analysis results, suggesting that the processing of phrase boundaries selectively activates more cortices, namely the superior temporal cortex, middle temporal cortex, medial orbitofrontal cortex, rostral middle frontal cortex, and rostral anterior cingulate cortex, before the occurrence of the stimuli.

**Table 1:** Statistics of significant temporal clusters in the comparison between iEEG sampled around note onsets and reference windows within each subregion in all twelve sessions.

Subregion	Number of temporal clusters	Mean of *p*-value	SD of *p*-value
Superior temporal cortex	21	0.010	0.012
Rostral anterior cingulate cortex	10	0.015	0.017
Rostral middle frontal cortex	14	0.024	0.015
Medial orbitofrontal cortex	11	0.014	0.016

**Table 2:** Statistics of significant temporal clusters in the comparison between iEEG sampled around phrase boundaries and reference windows within each subregion in all twelve sessions.

Subregion	Number of temporal clusters	Mean of *p*-value	SD of *p*-value
Middle temporal cortex	60	0.012	0.014
Superior temporal cortex	57	0.012	0.013
Rostral anterior cingulate cortex	63	0.011	0.013
Rostral middle frontal cortex	77	0.013	0.014
Medial orbitofrontal cortex	49	0.013	0.014
Superior frontal cortex	62	0.015	0.014
Insular cortex	61	0.012	0.014
Caudal middle frontal cortex	69	0.013	0.014

### Note-onset-related ERPs versus phrase-boundary-related ERPs

**Figure 8:**
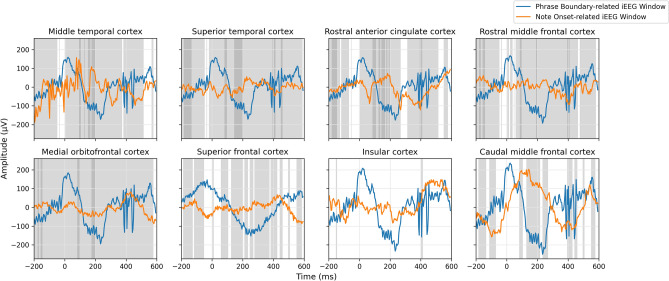
Note-Onset-related and Phrase-Boundary-related ERPs observed at these subregions with significant differences in at least six sessions are shaded in light gray, and ten sessions in dark gray respectively. Despite all note-onset-related iEEG windows being used in the analysis, eight windows were randomly sampled for display due to the large difference between the number of windows sampled around note onset and phrase boundaries and data variance.

Given that both note onsets and phrase boundaries elicited robust evoked responses, our next goal was to determine whether the brain processes these two stimuli differently by computing the ERP waveform (Fig. 8) and analyzing it with the cluster-based permutation test. The *p*-value statistics of significant temporal clusters are provided in Table 3.

**Table 3:** Statistics of significant temporal clusters in the comparison between iEEG windows sampled around phrase boundaries and iEEG windows sampled around note onsets within each subregion in all twelve sessions.

Subregion	Number of temporal clusters	Mean of *p*-value	SD of *p*-value
Middle temporal cortex	53	0.012	0.014
Superior temporal cortex	49	0.010	0.01
Rostral anterior cingulate cortex	58	0.012	0.013
Rostral middle frontal cortex	63	0.011	0.013
Medial orbitofrontal cortex	40	0.012	0.014
Superior frontal cortex	61	0.017	0.014
Insular cortex	53	0.012	0.014
Caudal middle frontal cortex	55	0.009	0.010

**Figure 9:**
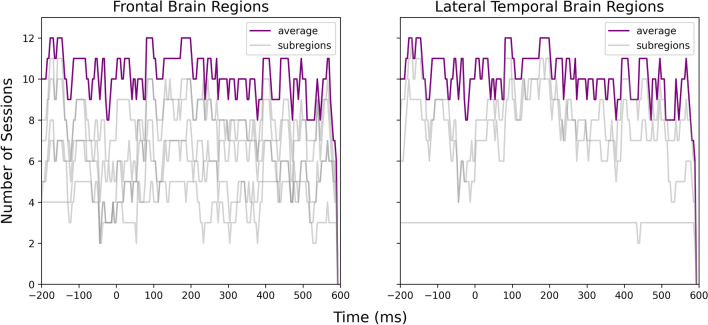
Number of sessions showing significant differences in the iEEG windows sampled around phrase boundaries and note onsets at each time point. The purple lines were obtained by averaging all channels within the frontal and lateral temporal brain regions. The light gray lines were obtained by averaging the channels within sub-regions that contributed to the frontal and lateral temporal regions.

**Figure 10:**
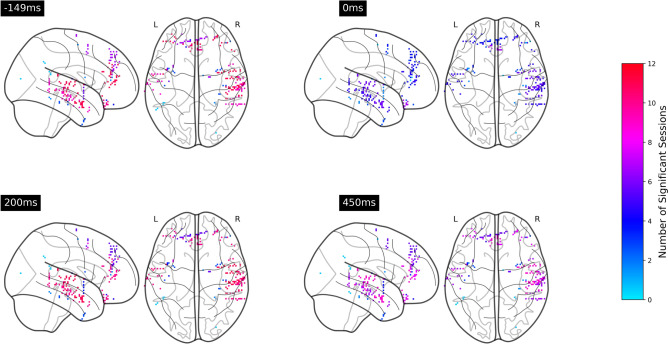
Number of sessions in which neural responses to note onsets and phrase boundaries are significantly different at $-149$, 0, 200, 450 ms relative to the stimulus markers .

**Figure 11:**
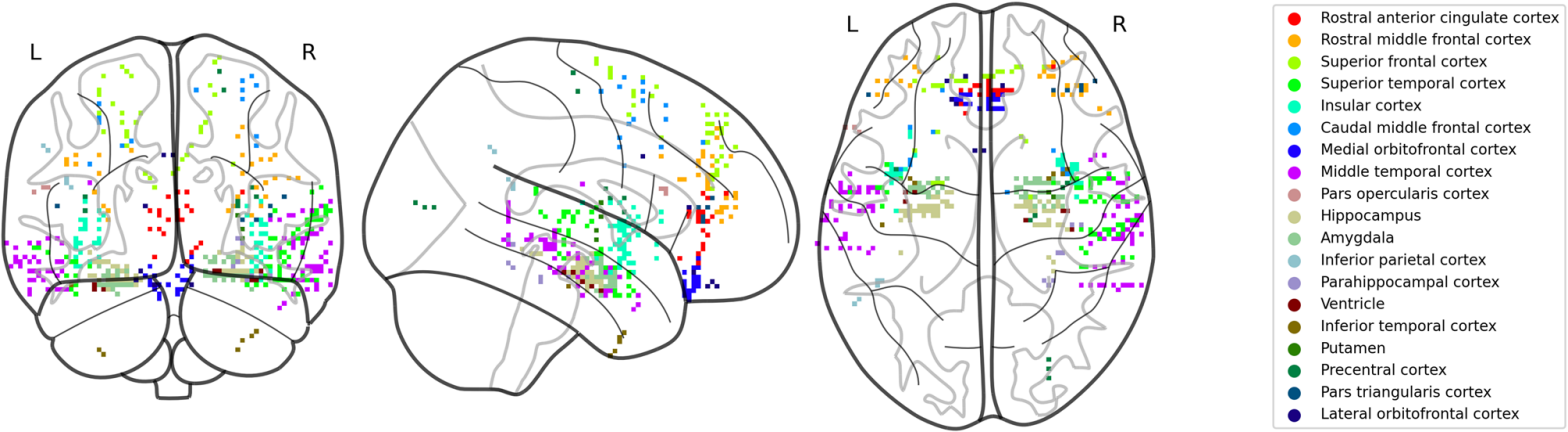
The location of electrodes within each subregion .

Figure 9 shows that the two ERPs were significantly different at around $-150$, 200 to 450 ms relative to the note onset and phrase markers in both frontal and temporal brain regions, with at least eleven sessions showing significant differences. Figure 10 further illustrates that the differences were mainly localized to the superior temporal cortex followed by the medial orbitofrontal cortex, rostral middle frontal cortex, and rostral anterior cingulate cortex. We also observed significant differences at $-150$, 100 to 200 and 400 to 500 ms relative to the stimulus markers in at least six sessions in the caudal middle frontal cortex, insular cortex, and superior frontal cortex (Fig. 11).

## Discussion

By integrating the results of within-session analysis, we examined whether note onsets and phrase boundaries elicited different neural responses across subjects. Several temporal clusters of significant difference were identified in the permutation test, demonstrating the difference between the neural responses to note onsets and phrase boundaries in terms of peak lag and amplitude. In addition to the auditory cortex, we were motivated to examine neural responses in frontal brain regions linked to grammatical structure building in studies of speech perception. Besides, the contrast between neural response to note-onsets and phrase boundaries in frontal brain regions may also reflect a process of building up syntactic structures with increasing hierarchy in music, similar to the computation merge in linguistics demonstrated by Zaccarella et al.^20^

We first confirmed that both note onsets and phrase boundaries elicited evoked responses by observing significant statistical differences between the iEEG windows sampled near the stimulus markers and the reference windows sampled between stimulus markers. Although we are the first intracranial EEG study to examine the evoked responses to note onsets and phrase boundaries using the cluster-level statistical analysis, our findings paralleled those of previous ERP studies using averaging techniques. A component was identified around 100 ms and 200 ms after the stimulus onset in both note-onset-related ERPs and phrase-boundary-related ERPs, which resembles the N1-P2 response in the auditory evoked response in language and music^21–24^. The N1-P2 like effect suggests that the processing of local cues takes place very quickly after the onset of the stimulus.

We then compared the neural responses elicited by note onsets and phrase boundaries and identified three temporal clusters at $-150$, 200, and after 400 ms relative to the stimulus markers with significant differences in at least eleven sessions.

An activation elicited by phrase boundaries at $-150$ ms was observed in both frontal and lateral temporal brain regions in all twelve sessions. The superior temporal cortex shows structural sensitivity in all twelve sessions, followed by the rostral middle frontal cortex, rostral anterior cingulate cortex, medial orbitofrontal cortex, and middle temporal cortex showing sensitivity in more than ten sessions. Although this prestimulus effect is non-significant in note-onset-related ERPs, we observed an activation of the rostral middle frontal cortex and medial orbitofrontal cortex in at least six sessions, which may reflect the entrainment of cortical rhythm to rhythm of the stimuli^25^. However, our analysis shows that this component is not consistent across sessions. The $-150$ ms component unique to the processing of higher-order structures was overlooked in earlier studies of phrase boundaries. The prestimulus activation in the superior temporal cortex could be interpreted by auditory attention, indicating the initiation of a new phrase which does not fit within the expectation of ongoing phrases^26^. The activation in frontal brain regions suggests a prediction response^27^, such as a reward effect of positive emotions resulting from anticipatory success. During exposure to music, participants gradually learned the information dynamics and were able to predict forthcoming phrase boundaries, due to changes in note density, melodic themes, key, tempo, and rhythm. This suggests that those neural representations which lead to correct predictions are strengthened and reused. This finding is in line with our previous study on the same dataset^28^, that an increased frontal theta power was observed during transitions from prolonged musical segments of Mozart’s K448 after at least 30-s exposure. The successful prediction of phrase boundaries may preferentially modulate activity in frontal emotional networks, suggesting that the widely observed strong pleasurable responses^29–31^ are linked to the prediction of higher-order musical structures.

Although N1-P2 like components were observed in both note-onset related ERPs and phrase boundaries related ERPs, the significant contrast between the two components, especially in the superior temporal cortex and middle temporal cortex with all twelve sessions showing significant differences, presumably reflects the processing of local cues mediated by more global expectation at phrase boundaries. The timing of the significant difference in the medial orbitofrontal cortex and rostral anterior cingulate cortex is also in line with an early negative component in frontal brain regions which is linked to the building of the grammatical structure in linguistic^32–34^.

The ERP components at 400 ms and 500 ms post-stimulus onset were only observed around phrase boundaries, potentially indicating higher-order feature extraction for processing the changes in the harmonic and rhythmic structure of the music. The 400 ms post-stimulus component has a broad scalp distribution, maximal in the superior temporal cortex, and is similar to the N400 response in timing thus possibly suggesting the conceptual processing in music^35^. However, this component is unlikely to be a music N400 response because we did not observe a clear negative-going wave as shown in the prior music N400 work^12^. The 500 ms component resembles CPS discussed in musical phrasing^18^. This CPS-like effect was observed in both frontal and temporal brain regions, maximal in the middle temporal cortex and superior temporal cortex. The activation in frontal brain regions suggests that these components may not only reflect the detection of phrase boundaries, but also a violation of melodic expectation in the transition from one phrase to the next. As shown in Fig. 13b, the first 90 s of Mozart’s K448 is structurally organized by contrasting melodic themes. The changes at phrase boundaries break the tension built up through harmonic and melodic progression within the previous phrases. Steinbeis et al.^36^ has reported that a violation of expectation could induce strong emotion. Huron^27^ further points out that an unexpected but innocuous event may result in anticipatory failure but generate positive emotions, known as the reaction and appraisal responses. Therefore, our findings were in line with the theory of musical expectations and emotion^37^.

**Figure 12:**
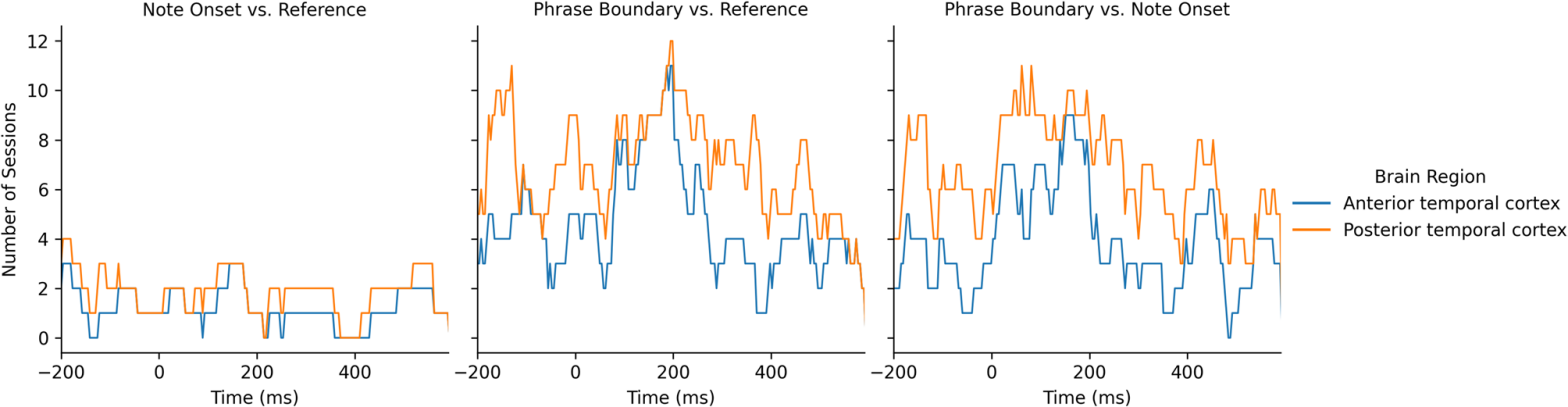
Number of sessions showing significant differences in the iEEG windows sampled in anterior temporal cortex and superior temporal cortex .

We also analyzed the neural response to note onsets and phrase boundaries in temporal regions as shown in Fig. 12. The posterior temporal regions showed a prestimulus effect on phrase boundaries but not note onsets, which is in line with recent works implicating the sensitivity of these regions in linguistics syntax processing^38–40^. Besides, the prestimulus effect was observed in posterior temporal regions but not anterior temporal regions, which suggests that this effect is more likely to be induced by music given that the posterior temporal regions are linked to the processing of pitch and temporal variation^41^.

The less significant findings of ERPs at note onsets were not unexpected. First, due to the high note density in a naturalistic music excerpt, the iEEG windows sampled around note onsets might cover multiple overlapped ERPs which could not be isolated because the intervals between note onsets were variant. These overlapped ERPs result in the non-significant peaks at $-150$ and 450 ms. Secondly, the randomly sampled reference windows might also contain ERPs elicited by weak note onsets which were excluded for comparison. To test this hypothesis, we compared the note-onset-related ERPs with iEEG windows randomly sampled during exposure to the silent washed-out period or violet noise. However, the experiment did not yield meaningful results. This might be explained by the yet unknown brain activities that the subjects undergo when not listening to music.

Our analyses extended Quon et al.’s study which shows that the musical structure of K448 may be contributing to its therapeutic effect^28^ and were performed on the same dataset on which Quon et al. observed a significant interictal epileptiform discharge (IED) reduction in bilateral frontal cortices coupled with increased frontal theta power during transitions from prolonged musical segments after at least 30-s of exposure to K448. It has been reported that listening to specific musical works, such as Mozart’s Sonata in D Major for Two Pianos (K448)^42–44^ and the Piano Sonata in C Major (K. 545)^45^, is associated with a reduction in seizure frequency and a reduction in abnormal interictal epileptiform discharges in patients with epilepsy. However, this effect has been demonstrated with only a small number of musical works with similar structures^46–48^, suggesting that this effect is dependent on musical structures such as a high degree of long-term periodicity^49,50^. In revealing the potential reward linked to prediction response occurring at phrase boundaries in Mozart’s K448, we shed light on the theory that structural organization of Mozart’s K448 could explain the mechanism behind music interventions such as the Mozart effect for epilepsy.

The results of our study must be interpreted in light of several limitations. First, we only studied the time-locked evoked and anticipatory responses while music perception also involves oscillatory response which could be estimated by an oscillator model. However, we considered oscillatory response to be trivial in our case because of the interplay between oscillatory and evoked components in auditory processing. Doelling et al.^51^ has shown that the evoked response can be reduced by smoothing the attack of note onsets. In contrast, the evoked response is the dominant response to the strong attack of note onsets that we investigated. Another major limitation was the overlapping of multiple note-onset-related ERPs within one window. Most importantly, we would like to acknowledge that the sample size might have limited our ability to generalize our results. The number of subjects was relatively small and 8 phrase boundaries were insufficient compared to 274 note onsets in the same music excerpt. Although previous studies^52^ have shown that 8 trials would be sufficient to detect certain ERP components, the statistical power does not saturate at this number. This could be improved in further studies by introducing more high-order structural changes in longer music excerpts.

In conclusion, our findings demonstrate that musical components at different hierarchical levels in Mozart’s K448 evoke consistent differential neural responses. We identify a prestimulus ERP component unique to note onsets occurring at musical phrase boundary, which indicates a predictive response in the frontal brain regions to higher-order structural changes within the music. These findings may guide future investigation of electrophysiological markers for processing hierarchy in music cognition and lead to new insights into potential auditory treatments for neurological disorders such as epilepsy.

## Material and methods

### Study population

A total of twelve sessions of Intracranial Stereo-EEG data were collected from eight subjects with refractory epilepsy undergoing monitoring for the clinical treatment. The electrodes were implanted based on clinical needs. These subjects had an average normalized baseline IED rate of 1.43 (SD 0.94). Each subject had electrode coverage in both hemispheres with between 34 and 77 artifact-free channels after excluding channels outside of MRI co-registered brain regions and bad channels for which the raw signal was greater than 2.5 standard deviations from the median value across channels. All subjects reported little to no previous musical training and limited exposure to classical music. Other subject demographic and clinical characteristics are provided in Table 4.

All patients provided informed consent to participate in this study, approved by the Committee for the Protection of Human Subjects (CPHS#: 12495) at Dartmouth College. Approval by CPHS was based on the study’s appropriate balance of risk and benefit to subjects and a study design in which risks to subjects are minimized. As such, our study followed the ethical standards laid down in the 1964 Declaration of Helsinki and its later amendments. Specific national laws were also observed, and all details that might disclose the identity of the subjects under study were omitted.

**Table 4:** Subject demographic. Left channels and right channels denote the number of contacts remaining after the exclusion of bad channels and channels outside of co-registered grey matter regions.

Subject	Gender	Age	Left channels	Right channels	Handedness
1	Female	29	68	0	Right
2	Female	43	0	25	Right
3	Male	30	32	18	Right
4	Male	56	44	45	Right
5	Male	27	51	51	Right
6	Male	27	51	38	Right
7	Male	32	2	38	Right
8	Male	35	60	0	Right

### Experiment paradigm

Each session of the experiment lasted approximately 30 minutes, consisting of 9 trials including (1) A baseline period only before the first trial of each session; (2) two minutes of a randomly sampled piece of music. The subject was required to finish the SART attention task, during the last 30 s of the music excerpt to confirm that the subject was attending to the piece of music. The attention task was reported separately; and (3) A washout period of one minute of silence after each music excerpt. Subjects listened to a 90-s violet noise and eight pieces of music including Mozart’s Sonata for Two Pianos in D major (K448) during data collection. The trials were repeated in random permutation until each piece of music was presented once. The current data analysis was only performed on sessions in which subjects listened to Mozart’s K448. Other auditory stimuli were Frederic Chopin’s Bolero in C–Op. 19 for piano, performed by Nikita Magaloff; Franz Liszt’s Piano Sonata in B Minor, 1st movement: Lento assai–Allegro energico, performed by Leslie Howard; Wagner’s Lohengrin Prelude to Act I; Mozart K448 with boosted 40Hz harmonics; and three songs chosen by each subject from a preferred musical genre (Tumbling Tumble Weeds by Sons of the Pioneers, Barbara Allen by Bradley Kincaid, Jugulator by Judas Priest, Just For by Nickelback, Na Na Hey Hey Kiss Him Goodbye by Steam, Peggy Sue by Buddy Holly). These eight auditory stimuli were excluded due to a lack of ground truth for phrase boundaries.

### Stimulus

**Figure 13:**
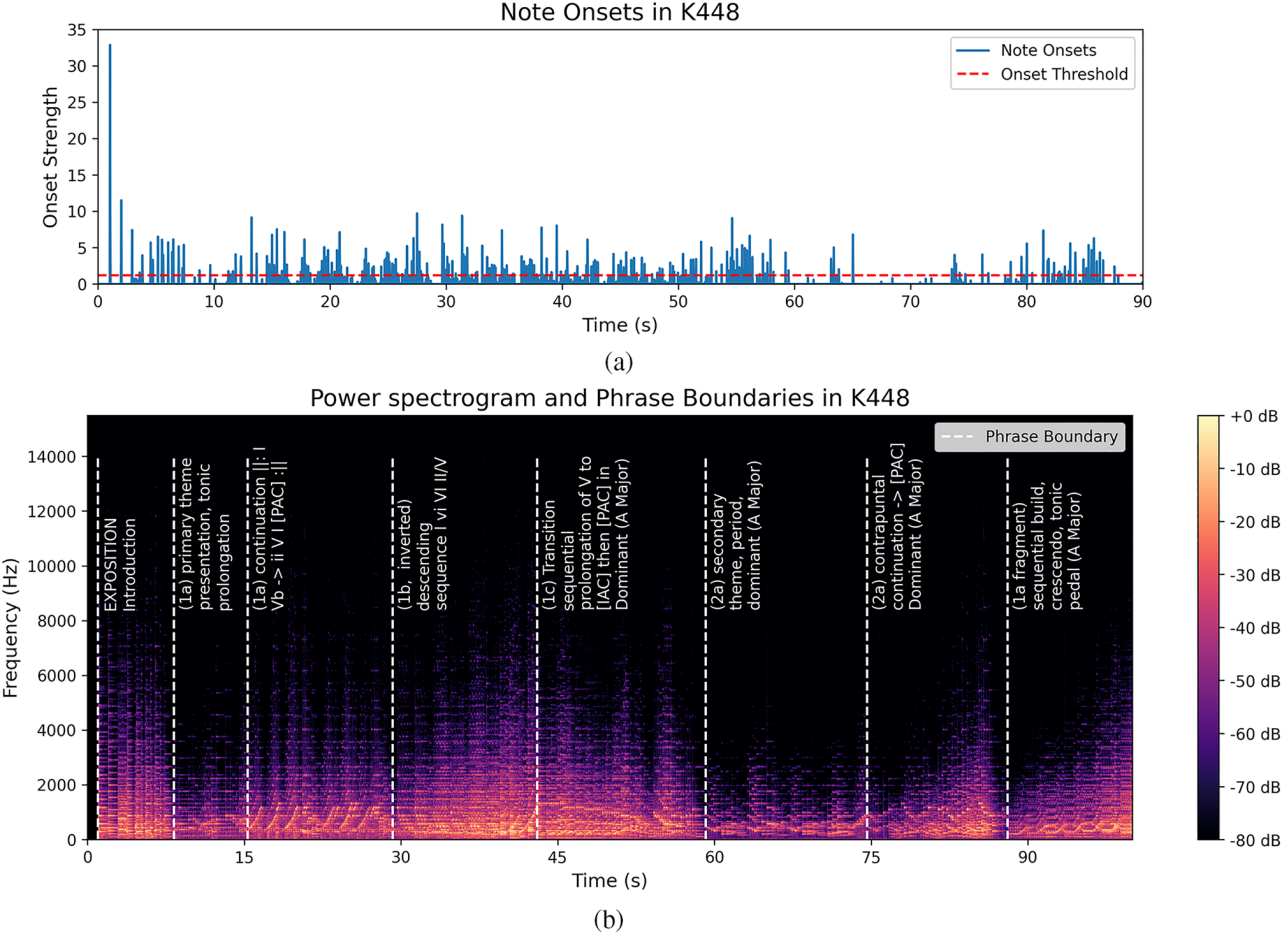
A visualization of the stimuli extracted from the first 90 s of Mozart’s K448. In (**a**), the blue lines mark the normalized note onset strength. 274 note onsets above the threshold marked by the red dotted line are selected. (**b**) shows the position of 8 phrase boundaries on the spectrogram of first 90 s of K448 .

Figure 13 shows 274 note onsets and 8 phrase boundaries extracted from the music excerpt as low-level and high-level musical components. The note onsets were detected by picking peaks in an onset strength envelope using librosa^53^. To reduce overlapping between iEEG windows sampled around two adjacent note onsets, we excluded $50\%$ of the weak note onsets based on the conclusion of previous studies^54,55^ that increasing stimulus intensity produces an increase in P300 amplitude of the ERP. The phrase boundaries were first annotated by a music expert on the score, and labeled in the audio by aligning the midi generated from the score with the audio using dynamic time warping (DTW). A theoretical evaluation of the first 90 s of Mozart’s K448 is performed to analyze the musical structure and annotated on Fig. 13b.

### Intracranial stereo-EEG data

iEEG was sampled at 512Hz from either 0.80-mm PMT platinum depth electrodes or 0.86-mm Ad-Tech platinum depth electrodes (Natus Medical Inc.). For all subjects, pre-implant T1-weighted and T2-weighted MRI images were co-registered with postoperative computed tomography (CT) to obtain the position of small-spacing Stereo-EEG depth electrodes. Freesurfer and the Desikan–Killany atlas were used for hippocampal subfield localization and cortical parcellation, and then final electrode positions were manually reviewed by two neuroradiologists^56–59^. The coordinates of the electrodes were transformed into a common MNI space for display. Figure 11 shows the electrodes placement within each subregion.

Due to the inconsistent electrode coverage across subjects and sessions, all the statistical analysis in this study was performed in a within-session manner.

The data were subsequently notch filtered at 60 Hz and band-pass filtered from 1 Hz to 250 Hz. All data were then re-referenced to an average referential montage, then downsampled to 256 Hz. This study was based on the data collected during exposures to the first 90 s of Mozart’s the Sonata for Two Pianos in D major, K448.

### Data segmentation

The iEEG data were segmented into windows around the stimuli. Each window started from 200 ms before the stimuli and 600 ms after the stimuli to include all desired ERP components. To generate reference windows for comparison in the analysis of note-onset-related and phrase boundaries-related ERPs, we randomly sampled 800 ms windows between note onsets with as little overlapping as possible. This resulted in 44 reference windows. The iEEG windows were then grouped by cortex and averaged across channels. The number of windows was resampled to 200 for statistical analysis.

### IED rejection

**Figure 14:**
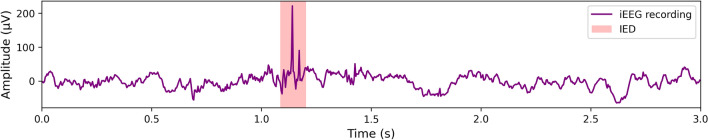
Intracranial recording from an electrode in the rostral anterior cingulate cortex of Subject 1 with an IED highlighted in red.

We rejected all iEEG windows that contained at least one interictal epileptiform discharge (IED) in at least one channel. The IEDs were detected using a template matching method^60^ which was validated and performed comparably to clinicians and other published detectors^61–64^. Figure 14 shows an example of an IED identified by this detector.

### Statistical analysis

To determine the most appropriate statistical measurement, we first examined the distribution of iEEG signal across channels at each time point within a window by normality test. Different tests were implemented based on the sample size of iEEG windows: D’Agostino and Pearson’s test was conducted for iEEG windows sampled around note onsets and reference windows; Shapiro-Wilk test was conducted for windows sampled around phrase boundaries windows. Since the data distribution at each time point was non-Gaussian, a two-sided Mann-Whitney U test was conducted in the statistical analysis.

We utilized a cluster-based permutation test^65^ to identify the consecutive temporal clusters in which neural responses were significantly different in two conditions and thereby verified the existence of note-onset-related and phrase-boundary-related ERPs. The cluster-based permutation test was conducted as follows: (1) A two-sided Mann-Whitney U test was performed at each time point within the window. The U statistics were converted to a time series of Z-scores. (2) The time points with Z-scores larger than the threshold were clustered based on temporal adjacency. The threshold was determined by the Z-score corresponding to the *p*-value of 0.05 in a two-sided test. (3) We repeated steps (1) and (2) on the data permuted for 1000 times if clusters were identified in step (2). The cluster-level statistics were calculated by taking the maximum of the Z-score within a cluster. The *p*-value of each cluster was given by the distribution of statistics on the permuted data. (4) We selected the temporal clusters with *p*-value $\le 0.05$.

## Data Availability

Deidentified Stereo-EEG data are available upon reasonable request.
